# The Role of Cilostazol, a Phosphodiesterase 3 Inhibitor, on Oocyte Maturation and Subsequent Pregnancy in Mice

**DOI:** 10.1371/journal.pone.0030649

**Published:** 2012-01-24

**Authors:** Min Li, Yang Yu, Jie Yan, Li-Ying Yan, Yue Zhao, Rong Li, Ping Liu, Aaron J. Hsueh, Jie Qiao

**Affiliations:** 1 Department of Obstetrics and Gynecology, Peking University Third Hospital, Beijing, China; 2 Program of Reproductive and Stem Cell Biology, Department of Obstetrics and Gynecology, Stanford University School of Medicine, Stanford, California, United States of America; 3 Beijing Key Laboratory of Reproductive Endocrinology and Assisted Reproductive Technology, Beijing, China; 4 Key Laboratory of Assisted Reproduction, Ministry of Education, Beijing, The People's Republic of China; State Key Laboratory of Reproductive Biology - Institute of Zoology Chinese Academy of Sciences, China

## Abstract

It is important to identify effective contraceptive drugs that cause minimal disruption to physiological processes. Phosphodiesterase 3 (PDE3) inhibitors suppress meiosis in oocytes by decreasing the level of cAMP and blocking the extrusion of the first polar body. In this study, we tested the PDE3 inhibitor, cilostazol, as a potential contraceptive agent. The effects of cilostazol treatment *in vitro* and *in vivo* on the suppression of oocyte maturation in a mouse model were investigated. The results indicated that treatment with increasing concentrations of cilostazol led to a dose-dependent arrest in meiosis progression. The effective *in vitro* concentration was 1 µM and was 300 mg/kg *in vivo*. The effect of cilostazol was reversible. After removal of the drug, meiosis resumed and mouse oocytes matured *in vitro*, and showed normal chromosome alignment and spindle organization. After fertilization using an ICSI method, the oocytes showed normal morphology, fertilization rate, embryo cleavage, blastocyst formation, and number of viable pups when compared with controls. The offspring showed similar body weight and fertility. *In vivo*, the mice became infertile if the drug was injected sequentially, and became pregnant following discontinuation of cilostazol. More importantly, no side effects of cilostazol were observed in treated female mice as demonstrated by blood pressure and heart rate monitoring. It is concluded that cilostazol, a drug routinely used for intermittent claudication, can effectively inhibit oocyte maturation *in vitro* and *in vivo*, does not affect the developmental potential of oocytes following drug removal and has few side effects in female mice treated with this drug. These findings suggest that cilostazol may be a potential new contraceptive agent that may facilitate an efficacy and safety study of this drug.

## Introduction

The current steroid-based contraceptive pills are reversible and effective. They also decrease the incidence of ovarian and endometrial tumors [1]. However, thrombogenic and other side effects in some women taking these steroid-based contraceptive pills have been reported [2], and the effects of these drugs on future generations are still unclear. The ideal contraceptive should act on the ovary to block oocyte maturation during the process of ovulation without disrupting the menstrual cycle.

In mammals, oocyte meiosis is arrested at the germinal vesicle (GV) phase in growing follicles. Before ovulation, the LH surge induces granulosa cells to secrete cyclic adenosine monophosphate (cAMP) which leads to follicle rupture [3], [4]. However, in oocytes, a decrease in intra-oocyte cAMP levels is required for the resumption of meiosis. Earlier studies demonstrated that spontaneous maturation of mouse oocytes *in vitro* when released from their follicles can be reversibly blocked by the addition of a derivative of cAMP or a phosphodiesterase (PDE) inhibitor and the PDE3 was localized in oocytes [5]. Treatment with a PDE3 inhibitor did not affect follicle rupture and reproductive cyclicity in mice but elevated cAMP levels in oocytes and suppressed GV breakdown, leading to a new contraceptive approach [6]. Thus, by increasing the level of cAMP with pharmacological or molecular approaches, one can inhibit meiosis in oocytes and induce contraception.

To date, ORG9935 is one of the widest studied PDE3 inhibitors as a potential contraceptive. Oocytes retrieved from immature follicles were arrested in prophase I with a high efficiency for up to 72 h when cultured with ORG9935 (10 mM) [7]. A series of experiments on macaques found that ORG9935 selectively blocked the spontaneous resumption of meiosis in macaque oocytes *in vitro* and inhibited oocyte maturation in gonadotropin-stimulated and in natural ovarian cycles in rhesus macaques without affecting follicle rupture [8]–[10]. The effective dosage of ORG9935 for oocyte meiotic arrest in rhesus macaques was also determined [11]. It has been proposed that this PDE3 inhibitor could be a potential oral contraceptive. However, treatment with ORG9935 in rodents increased heart rate [6] and ORG9935 is not a clinical medicine approved by the US Food and Drug Administration (FDA). Therefore, it is still necessary to develop other PDE3 inhibitors approved by FDA and study their potential as contraceptives.

Cilostazol, 6-[4-(1-cyclohexyl-1H-tetrazol-5-yl)butoxy]-3,4-dihydro-2-(1H)-quinolinone, another PDE3 inhibitor, has been shown to increase cellular levels of cAMP by inhibiting its degradation, it also inhibits platelet aggregation [12], serving as an arterial vasodilator. Importantly, it has been approved as a therapeutic agent for intermittent claudication [13]. However, the possible regulation of oocyte maturation *in vitro* and *in vivo* by cilostazol has yet to be studied.

The aim of this study is to investigate the effects of cilostazol on meiotic arrest and maturation of mouse oocytes *in vitro* as well as the potential use of cilostazol as a contraceptive *in vivo* in mice. The effects of prior exposure to cilostazol on chromosome alignment and spindle organization in oocytes, development during pregnancy and full-term development of embryos after removal of cilostazol were also studied. Furthermore, the physiology indexes of mice treated with this drug and their offspring was investigated.

## Results

### Effects of different concentrations of cilostazol on the maturation of mouse oocytes *in vitro*

**Figure 1:**
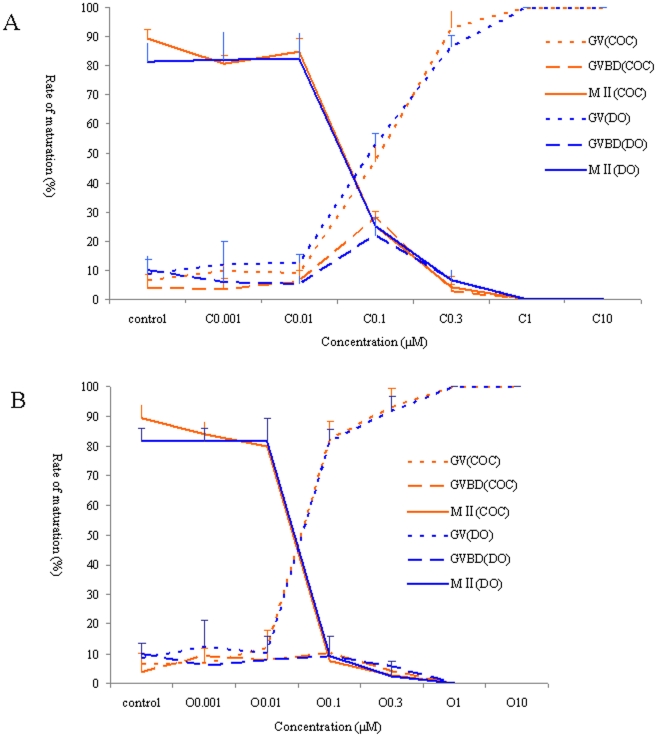
Developmental stages of mouse oocytes after treatment of COCs and DOs with cilostazol and ORG9935 at different concentrations for 24 h. As the concentration increased, the percentage of oocytes showing meiotic arrest increased (*P*<0.05). (A) Cilostazol and (B) ORG9935. Orange dot line represents COCs at GV stage, orange hyphen line represents COCs at GVBD stage, and orange straight line represents COCs at MII stage. Blue DOs dot line represents COCs at GV stage, blue hyphen line represents DOs at GVBD stage, and blue straight line represents DOs at MII stage.

To study the optimal concentration of cilostazol for the inhibition of mouse oocyte maturation, different concentrations of cilostazol were added to the IVM medium containing mouse cumulus-oocyte complexes (COCS) and denuded oocytes (Dos). A dose-dependent suppression of meiotic progression was observed (Fig. 1A). With increased concentrations of cilostazol, the meiotic arrest rate of GV oocytes was also increased, and meiotic arrest was completely inhibited at 1 µmol/L. The effects of different concentrations of ORG9935 on mouse oocytes were also evaluated as a positive control and were consistent with those found in cilostazol treatment (Fig. 1B).

### Reversible effects of cilostazol on the developmental competence of oocytes and embryonic development

**Figure 2:**
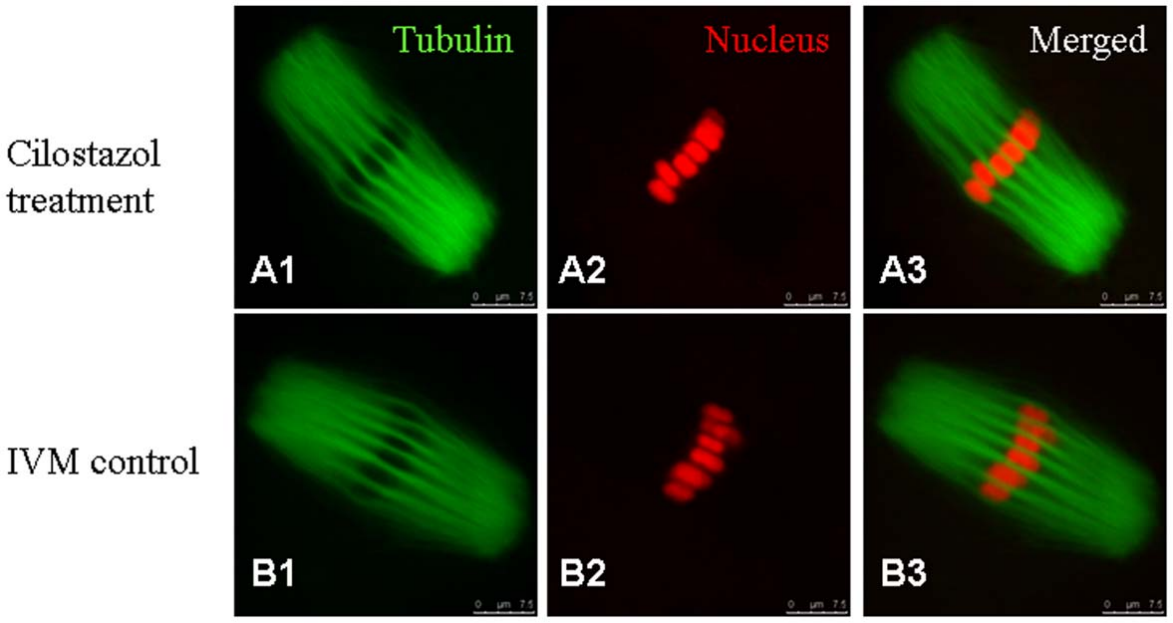
The distribution and organization of microtubules and spindles in IVM oocytes treated and not treated with cilostazol. (A) Representative normal spindle and chromosome alignment in the treatment group. (B) Representative normal spindle and chromosome alignment in the non-treatment group. Original magnification ×400.

Following removal of cilostazol, meiosis resumed within 20 hours which is normal in IVM. Some of the IVM oocytes which recovered from meiotic arrest were tested for spindle organization and chromosome alignment. The results showed that there was no significant difference between IVM oocytes treated with cilostazol and control IVM oocytes (P>0.05). More than 80% of IVM oocytes in the treatment and control groups showed normal spindle organization and chromosome alignment, and spindle configuration was regarded as morphologically normal when the meiotic spindle showed an oblong structure. Chromosome configuration was regarded as normal when chromosomes were located exclusively on the equatorial plate (Table 1). The representative morphology is shown in Fig. 2.

**Table 1:** A summary of α-tubulin expression and nuclear location in IVM oocytes in cilostazol treatment and non-treatment groups.

Group	No. of oocytes	Normal Metaphase	Abnormal Metaphase
Treatment	22	16 (72.7)^a^	6 (27.3)^a^
Non-treatment	28	21 (75.0)^a^	7 (25.0)^a^

a Values with same superscripts in the same column are comparable (P.0.05).

Some of the IVM oocytes were fertilized using the ICSI method to assess the developmental potential of the oocytes treated with cilostazol. Successful fertilization was assessed by the formation of male and female pronuclear. Some fertilized embryos were cultured *in vitro* to assess the preimplantation development potential. The rates of fertilization and cleavage *in vitro* were 78.3% and 83.1%, respectively, which were not significantly different from the control group (P>0.05). The rate of blastocyst formation was also comparable between the two groups, showing no significant difference (P>0.05) (Table 2). Other fertilized embryos were transferred into female recipients to assess full-term development potential. The results suggested that there was no significant difference between the treatment and control groups (P>0.05) (Table 2).

**Table 2:** Effects of cilostazol treatment *in vitro* and *in vivo* on the early and full-term development of ICSI embryos.

Group	No. of oocytes	No. fertilized (%)	Development stage (%)			No. of transferred embryos	No. of full-term fetuses (%)
			2-cell	8-cell	Blastocyst		
Treatment *in vitro*	83	65 (78.3)^a^	54 (83.1)^a^	33 (64.1)^a^	23 (42.6)^a^	30	14 (46.7)^a^
Control *in vitro*	77	62 (80.5)^a^	50 (80.6)^a^	30 (60.0)^a^	18 (36.0)^a^	30	16 (53.3)^a^

a Values with same superscripts in the same column are comparable (P.0.05).

**Figure 3:**
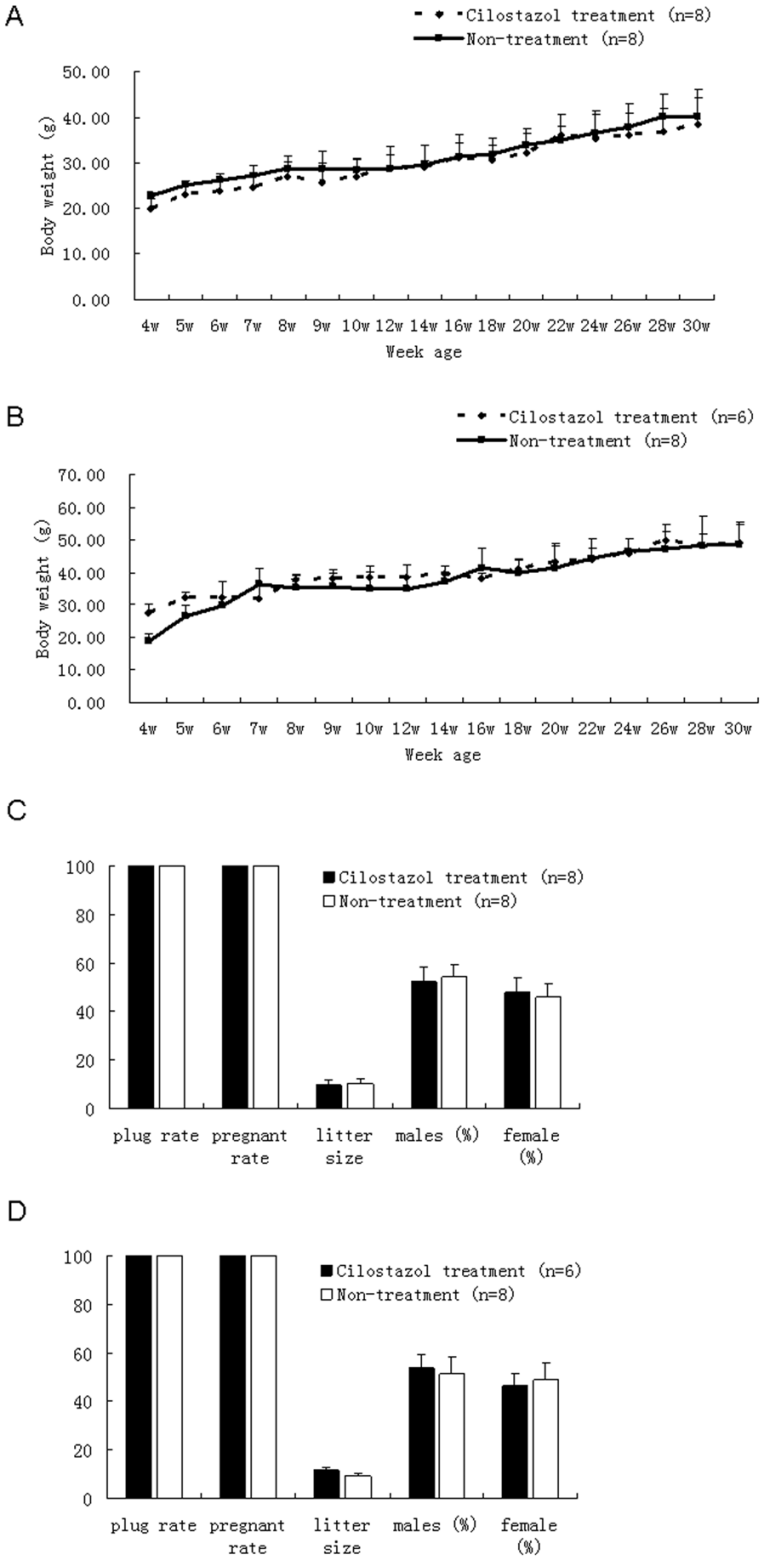
Measurements of body weight and fertility in offspring from oocytes treated and not treated with cilostazol. (A) body weight of female mice, (B) body weight of male mice, (C) fertility of female mice, (D) Fertility of male mice. The value of the litter size is not a percentage, but numerical data. In image (A) and (B), Black hyphen line with diamond represents mice conceived from female mice treated with cilostazol before, and black straight line with square represents mice conceived from female mice without cilostazol treatment. In image (C) and (D), Black solid rectangle represents cilostazol treatment group, and black hollow rectangle represents control group without cilostazol treatment.

Two key physiological indices of offspring were analyzed: body weight and fertility. The results of body weight analysis indicated that there was no significant difference between female and male offspring in the treatment and control groups (P>0.05). However,, the body weight of male offspring in the seven-week and sixteen-week age in the treatment group was significant lower compared with the control group (P<0.05) (Fig. 3A and B). The fertility assessment demonstrated that there were no significant differences between the treatment and control groups (P>0.05), irrespective of when the female or male mice were mated. No significant differences in litter size and sex ratio were observed between the two groups (P>0.05) (Fig. 3C and D).

### Effects of different concentrations of cilostazol on the oocyte maturation, pregnancy and heart cardiovascular parametes in female mice

**Figure 4:**
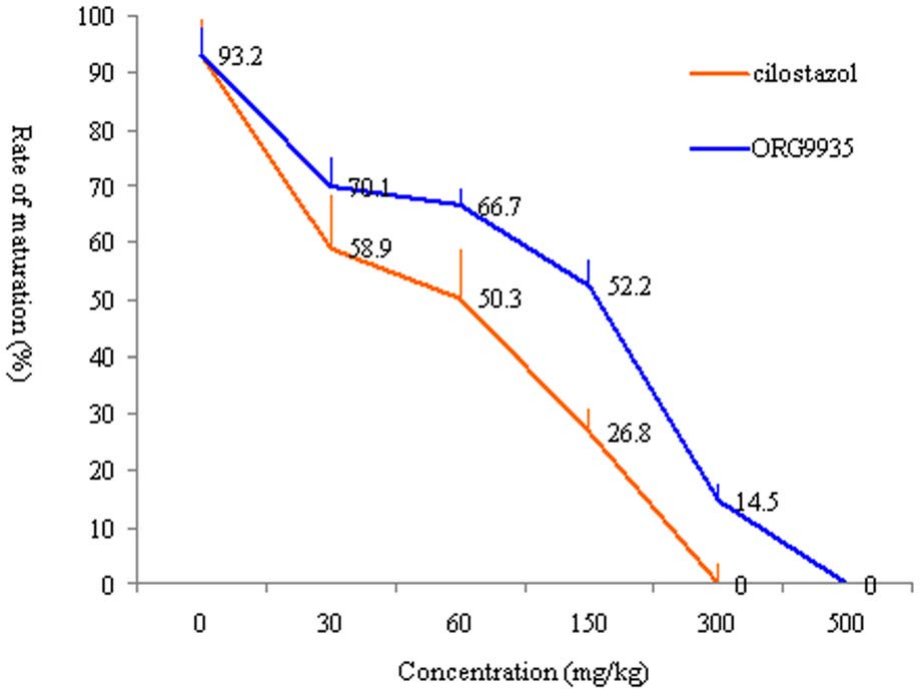
Effects of *in vivo* treatment with different concentrations of cilostazol and ORG9935 on the GVBD ratio of oocytes in mice. Orange straight line represents cilostazole treatment group, and blue straight line represents ORG9935 treatment group.

Based on *in vitro* studies, we injected cilostazol into female mice to evaluate its role in the suppression and inhibition of oocyte maturation and pregnancy. Mice treated with ORG9935 acted as positive controls. The results showed that cilostazol had a dose-dependent effect on *in vivo* maturation of oocytes. With increased concentrations of cilostazol, the rate of mature MII oocytes was decreased, and meiotic arrest was completely inhibited at 300 mg/kg, and approximately 14.5% of oocytes treated with ORG9935 were not inhibited and achieved maturation, the effective concentration of ORG9935 was 500 mg/kg (Fig. 4).

**Figure 5:**
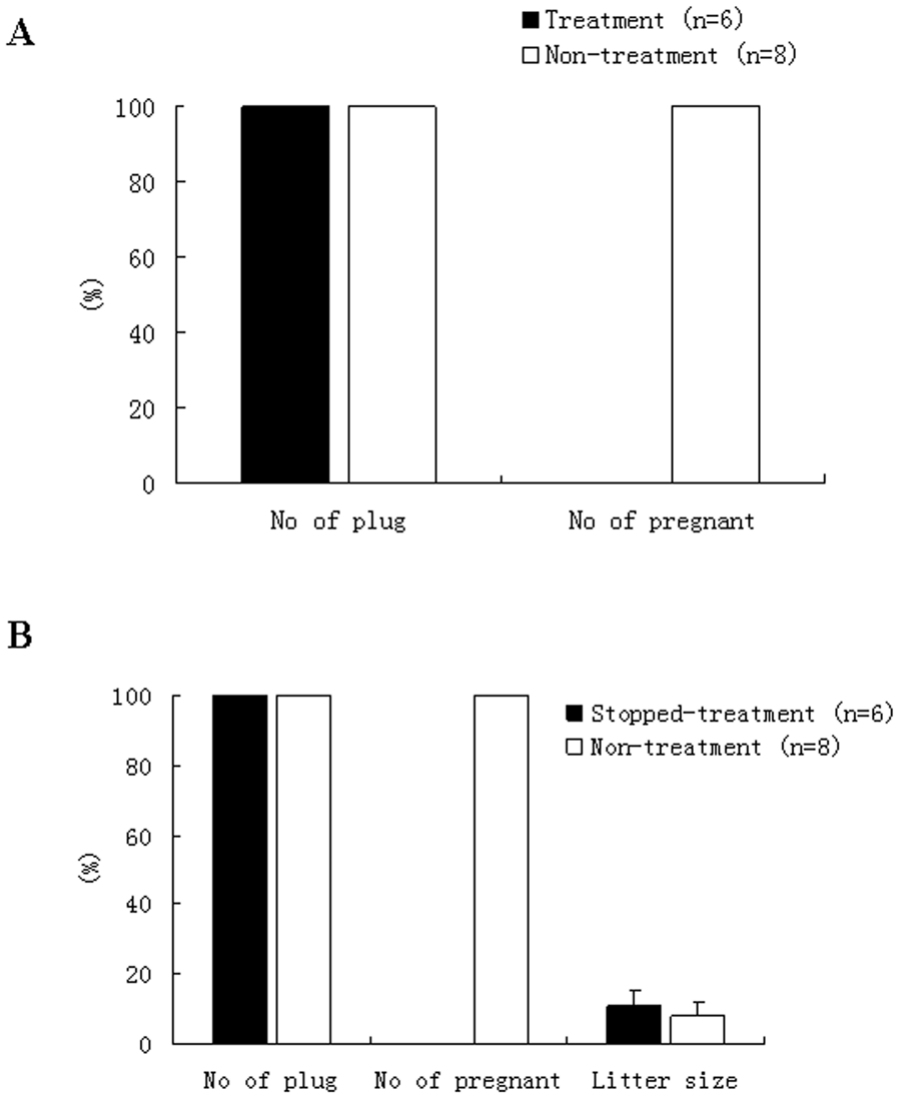
Effects of cilostazol administration on the fertility of female mice. (A) treated with cilostazol, (B) treatment with cilostazol stopped. The value of the litter size is not a percentage, but numerical data. In image (A), Black solid rectangle represents cilostazol treatment group, and black hollow rectangle represents control group without cilostazol treatment. In image (B), Black solid rectangle represents cilostazol treatment group, but stopped treatment at present, and black hollow rectangle represents control group without cilostazol treatment.

Female mice treated with the cilostazol were mated with male mice. The results suggested that the mice injected consecutively with cilostazol did not become pregnant although vaginal plugs were found in the morning. However, mice injected with the same volume of 0.9% physiological saline solution became pregnant and produced offspring after plugs were observed. Following the removal the cilostazol, fertility was restored in female mice within a short time, they became pregnant and produced offspring, and the litter size in treated mice was not significantly different from that in non-treated mice (Fig. 5A and B).

To identify possible side effects of cilostazol in female mice, the blood pressure and heart rate of female mice injected with cilostazol were monitored. The results showed that heart rate was not significantly different between the treatment and control groups (P>0.05), and the results for systolic and diastolic blood pressure (SBP and DBP) were similar (Table 3).

**Table 3:** Comparison of heart rate and blood pressure in mice before and after cilostazol administration (Mean ± S.E.M).

Groups	Blood pressure		Heart rate
	SBP (mmHg)	DBP (mmHg)	
Before treatment	105.475±4.134^a^	67.788±4.058^a^	367.315±31.459^a^
After treatment	108.100±4.375^a^	70.075±4.200^a^	395.949±33.750^a^

a Values with same superscripts in the same column are comparable (P.0.05).

## Discussion

In the present study, we first described the role of cilostazol, a PDE3 inhibitor, on the suppression of mouse oocyte maturation *in vitro* and *in vivo*, and demonstrated the effects of cilostazol on IVM oocytes and the resulting embryos and offspring. PDE are enzymes that can degrade and inactivate cAMP [14]. PDE3 is a member of the PDE family, and is found in the oocytes of mice [15], cattle [16], and humans [7]. Inhibition of PDE3 can increase the level of cAMP, resulting in oocyte GVBD blockage [16]–[25]. PDE3A regulates the resumption of meiosis up to 3 h prior to GVBD and transiently affects meiotic progression [16], [26]. PDE3A-deficient mice with oocytes containing increased cAMP levels failed to undergo spontaneous maturation; however, the animals were viable and showed no other abnormalities [26]. Adult cycling rats treated with a PDE3 inhibitor completely prevented viable pregnancy, but maintained estrous cycles (and ovarian hormone production) [6].

Our study showed that cilostazol effectively inhibited mouse oocyte maturation *in vitro* and *in vivo*. These results may provide a potential new approach for future contraceptives. The effective concentration of cilostazol was identified by *in vitro* and *in vivo* experiments using a mouse model, and reversibility tests indicated that the developmental competence of the oocytes was not impaired following removal of the drugs and allowed ovulation and oocyte maturation. In the *in vitro* experiments, ORG9935 suppressed meiosis at the concentration of 1 µmol/L, consistent with the findings of a previous study [16].

It is a concern for users as to whether the developmental competence of oocytes would be impaired by this drug. Using the mouse model, the dynamics of the spindle and chromosome apparatus were identified, and the results suggested that there was no significant difference between the treatment and control groups. Moreover, the resulting fertilized embryos had similar development potential in the preimplantation and full-term development stages as those in the control group. In our study, treatment with cilostazol did not affect the development potential of treated oocytes after drug removal, Similarly, mouse follicles treated with ORG9935 in IVM medium did not affect somatic cell function, differentiation, or oocyte growth and maturation [27].

In the present study, we found that cilostazol was safer than ORG9935. Wiersma et al. indicated that ORG9935 could induce an increase in heart rate in rodents. However, in our study, the heart rate in female mice treated with cilostazol was normal and not significantly different to that in the control group. The mating experiment was used to assess the fertility of mice treated with this drug and to determine the safety of this drug. The mice treated with cilostazol were infertile, and immediately became pregnant after its removal. The litter size and sex ratio of the offspring from the treated oocytes and normal IVM oocytes were similar. All these data demonstrate that cilostazol is effective and safer than the PDE3 previously reported.

Furthermore, cilostazol has been approved in the U.S. for the treatment of symptoms of intermittent claudication (IC) since 1999 and for related indications since 1988 in Japan and other Asian countries [28]. Other PDE3 inhibitors such as milrinone have been described as having side effects such as fatal arrhythmias, however, no such side effects have been described for cilostazol [29]. Cilostazol has also been confirmed to be beneficial in different circulatory symptoms [12], [30], [31]. If the correct dosage is identified to minimize side effects, this agent may be a safe contraceptive.

In conclusion, we found that cilostazol blocked maturation of mouse oocytes both *in vitro* and *in vivo*. The reversibility of this drug was determined based on the resumption of oocyte meiosis and blastocyst formation as well as full-term development of fertilized embryos. The effectiveness of cilostazol was proved by the mating experiment, which indicated the potential of cilostazol as a contraceptive drug. These findings may not only provide a new choice of contraceptive, but may promote and facilitate future studies on the mechanisms of action of contraceptive drugs on oocyte and embryo development.

## Materials and Methods

All chemicals were from Sigma Chemical Co. (Sigma, USA) unless otherwise indicated. ORG9935 and cilostazol were stored at 10 mM in dimethyl sulfoxide (DMSO) at −20°C. In preliminary experiments, the highest concentration of the DMSO carrier (0.1% v/v) was tested for 24 h in IVM medium and no effect on maturation was observed.

### Experimental design

A schematic diagram of the study is shown in Figure S1. Experiment 1 was primarily designed to determine the lowest effective dose of cilostazol that arrested oocytes *in vitro* at the GV-stage with full reversibility of the inhibitory effect. Different concentrations were tested: 0 (control), 0.001, 0.01, 0.1, 0.3, 1 and 10 µM. After 24 h of incubation with cilostazol, groups of cumulus-oocyte complexes (COCs) were denuded, and were classified by light microscopy as GV, GV breakdown (GVBD) or polar body extrusion (PB). Experiment 2 determined the spindle organization and chromosome alignment of IVM oocytes following removal of cilostazol, and the developmental potential of the resulting embryos fertilized by the ICSI method in the early stage of development and at full-term. Body weight and reproductive ability in offspring were also analyzed. Experiment 3 was an *in vivo* experiment which assessed whether cilostazol affected the fertility of mice, and the side effects induced by this drug.

### Mice and collection of oocytes

ICR inbred mice (CD-1, Animal Center of Medical College of Peking University), at 8–10 weeks of age were housed and bred in the Animal Center of Medical College of Peking University according to national legislation for animal care. Mice at the proestrous stage were injected intraperitoneally with 10 IU pregnant mare serum gonadotropin (PMSG) and sacrificed 48 h later by cervical dislocation in order to obtain COCs and denuded oocytes (DOs) from small antral follicles. The ovaries were dissected and collected in Leibovitz-15 medium, supplemented with 10% fetal calf serum (FBS, GIBCO), 100 mg/ml streptomycin, and 100 IU/ml penicillin. To prevent spontaneous resumption of meiosis, cilostazol or ORG9935 was added to this medium at the same final concentration as used in the IVM medium.

COCs and DOs were isolated mechanically by puncturing antral follicles with fine needles. Only DOs with a GV and COCs consisting of an oocyte surrounded by a compact cumulus-cell mass were selected for the experiments.

For *in vivo* experiments, oocytes were obtained by priming mice with 10 IU/ml PMSG followed at 48 h by an injection of 10 IU human chorionic gonadotropin (hCG). Two hours before injecting hCG, mice were given ORG9935 or cilostazol by feeding at concentrations of 30 mg/kg, 60 mg/kg, 150 mg/kg, 300 mg/kg, and 500 mg/kg. After sacrificing the mice, COCs were obtained from the oviduct. To assess oocyte development, we used 80 IU/ml hyaluronidase to remove the cumulus cells surrounding the oocytes.

### *In vitro* maturation of oocytes

Immediately after aspiration, the oocytes were washed and placed into 15 µl droplets containing different concentrations of the drugs overlaid with oil. The IVM medium (Invitrogen, Sweden) was prepared the day before and supplemented with 10 U/L FSH and LH. The drugs were added to the IVM medium before oocyte retrieval. All cultures were carried out at 37°C in a humidified atmosphere in an incubator gassed with 5% CO_2_ in air. At 24 h of culture, the stages of oocyte development were recorded and oocytes with PB I were removed from culture. Oocytes were denuded mechanically by repeated pipetting and were assessed for maturation by scoring for GV, GVBD and PB1.

### Immunofluorescence staining and confocal analysis

Mature (IVM) oocytes were fixed with 4% paraformaldehyde in phosphate-buffered saline (PBS) *in vitro* for 30 min at room temperature. They were then permeabilized in incubation buffer (1% Triton X-100 in PBS) for 15 min at 37°C in an incubator. This was followed by blocking in 1% BSA for 1 h and overnight incubation at 4°C with rabbit anti-α-tubulin antibody (1∶100) for spindle staining. After three washes in PBS with 0.1% Tween-20 and 0.1% Triton X-100 for 5 min, the oocytes were incubated with FITC-conjugated goat anti-rabbit IgG (1∶100, Santa Cruz biotechnology, Inc, US) at 37°C for 1 h. The oocytes in the control group were only incubated with the FITC-conjugated secondary antibody. The oocytes were then incubated with PI at room temperature for 15 min. The oocytes were observed using a laser scanning confocal microscope (LSCM; Biorad Radiance 2000 mounted on a Nikon inverted microscope; Tokyo, Japan) to analyze spindle size, shape and chromosome alignment.

### Intracytoplasmic sperm injection, embryo culture and embryo transfer

After removal of the drugs at 1 µmol/L, the oocytes were cultured in IVM medium for 20 h and MII oocytes were used for ICSI. Sperm collected from the cauda epididymis of adult ICR mice was washed twice with injection buffer (75 m mol/L KCl and 20 m mol/L HEPES, pH 7.0), then treated with buffer containing 12% polyvinylpyrrolidone. Active sperm was delivered into the oocyte cytoplasm using a Piezo micropipette-driving unit as described [32]. A slight piezo-electric pulse was applied to puncture the oocyte plasma membrane following penetration of the zona pellucida. Mouse sperm heads were separated from tails by applying a few piezo-pulses at the mid-piece of the sperm, immediately prior to injection into the oocyte. Activation was assessed by the number of zygotes with extrusion of the second PB and two pronuclei at 6 h post-ICSI, and cleavage to the 2-cell stage after 24 h. *In vitro* development of embryos was assessed by monitoring progression to the blastocyst stage on day 4.

After ICSI manipulation, the embryos showing two-pronuclei (2PN) were transferred to the oviduct of pseudopregnant CD-1 females and the pregnant mice delivered naturally on day 19.5. The offspring were maintained under controlled temperature and lighting conditions, and given food and water ad libitum. Postnatal development of mice was evaluated according to a panel of physiological indices, including body weight and reproductive ability.

### Body weight

The offspring were weighed weekly before 10 weeks and once every two weeks after 10 weeks.

### Measurement of blood pressure and heart rate

Individual mice were placed in a cage on the warmplate at 37°C for half an hour. Then systolic blood pressure (SBP) and heart rate (HR) were measured by the tail-cuff method with a Softron BP system (Softron BP-98A, Tokyo, Japan). The measured mice were put back into the cage to rest, then injected with 300 mg/kg cilostazol, and allowed to calm down in the cage on the warmplate at 37°C for 2 h. SBP and heart rate (HR) were then measured by the tail-cuff method with a Softron BP system.

### Fertility studies

Female mice receiving cilostazol were mated, and the vaginal plugs were checked every morning during the following two weeks. Female mice with plugs were separated and fed individually in cages.

In the offspring, the female and male mice were mated, and the pregnant mice were separated and fed individually in cages. The litter size and the sex ratio were recorded.

### Statistical analysis

Experiments were performed at least three times and *P*≤0.05 was considered statistically significant. The values are given as means ±S.E.M. For the data on oocyte maturation, n refers to the number of oocytes. Maturation frequencies were subjected to arcsine transformation and analyzed by ANOVA followed by Duncan's multiple range tests.

## Supporting Information

Figure S1 **A schematic diagram of the study.**
(TIF)
