# Supplementary figures and images for: The Role of Cilostazol, a Phosphodiesterase 3 Inhibitor, on Oocyte Maturation and Subsequent Pregnancy in Mice
Source: PLoS One. 2012 Jan 24;7(1):e30649. doi: 10.1371/journal.pone.0030649 (PMC3265514; doi:10.1371/journal.pone.0030649)

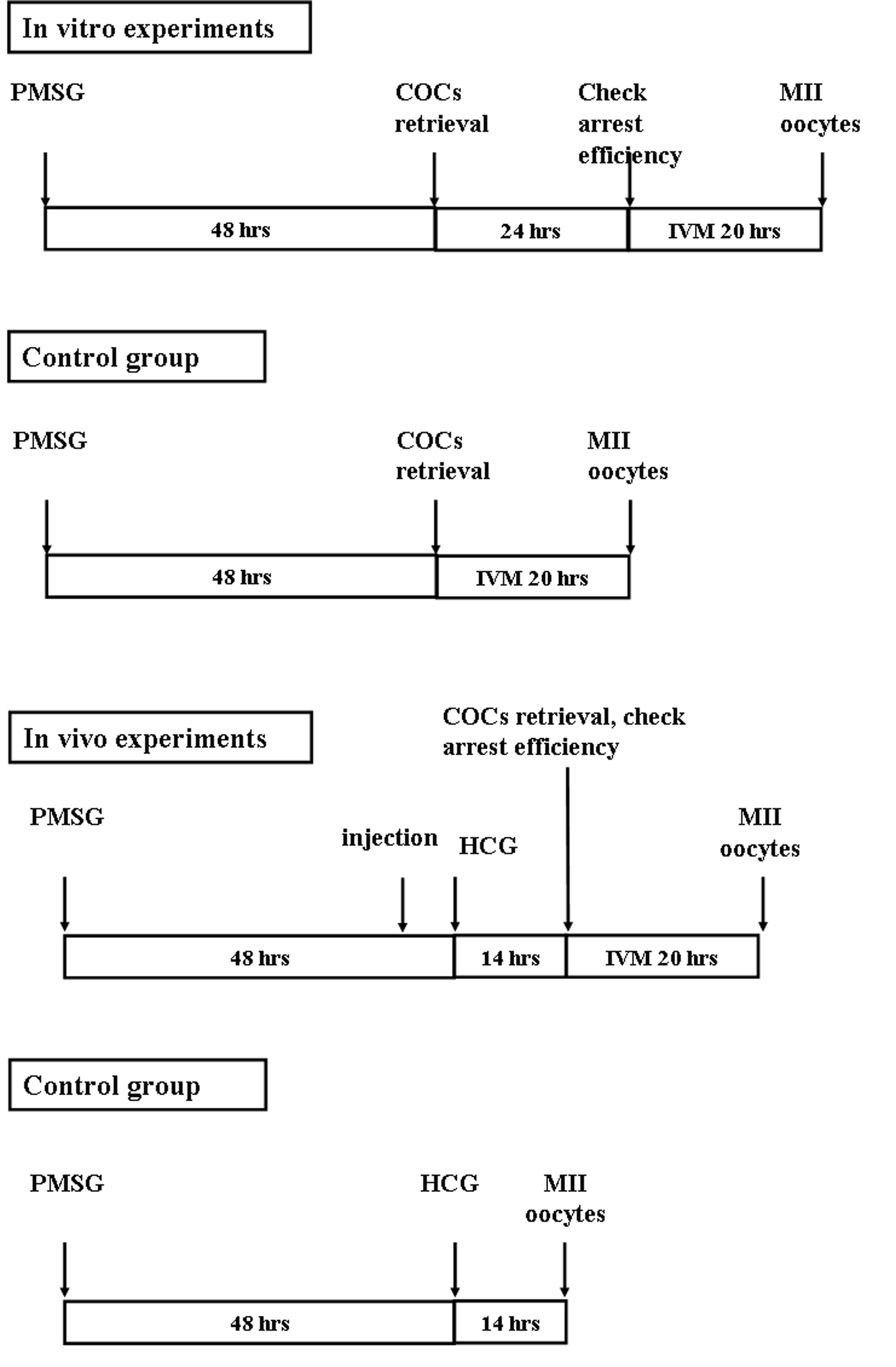

Supplement: Figure S1 — A schematic diagram of the study. (TIF) [file pone.0030649.s001.tif]
